# A clinical case of anosognosia in a CADASIL disease.

**DOI:** 10.1192/j.eurpsy.2023.2125

**Published:** 2023-07-19

**Authors:** E. Talaya Navarro, L. Gallardo Borge, E. Gómez Fernández, R. Fernández Díaz, L. Al Chaal Marcos, E. Rybak Koite

**Affiliations:** Psiquiatría, Hospital General de Segovia, Segovia, Spain

## Abstract

**Introduction:**

CADASIL (Cerebral Autosomal Dominant Arteriopathy with Subcortical Infarcts and Leukoencephalopathy) is a cerebrovascular disease, tht appears in 1.98/100,000. It´s caused by a mutation of the Notch3 gene and is characterized by accumulation of granular osmiophilic material in the middle layer of the small and median sized cerebral arteries.

Sypmtoms are migraine, recurrent cerebral ischemic episodes, dementia, neuropsychiatric disorders (anosognosia, character disorders, apathy and cognitive impairment). It usually appears between 30-60 years, although there is an important variability. There is no curative treatment, only palliative.

**Objectives:**

Clinical review of anosognosia and its presence in CADASIL disease.

**Methods:**

Clinical case and literatura review.

**Results:**

We presented the clinical case of a 68-year-old man, who was diagnosed with CADASIL after a stroke 3 years earlier. In his family, his brother was diagnosed also with CADASIL. The patient had previously presented disturbances in impulse control (hyperorality) and important executive failures. He currently presented anosognosia, deficits in verbal memory, spatial perception and executive functions, in addition to behavioral alterations and apathy. Due to these deficits he was prohibited from certain activities (driving, hunting).

The patient was not aware of these deficits and becouse of his “no knowledge of his illness”, he disagreed with these prohibitions, so he showed rage and anger at the impotence of not understanding why certain actions are prohibited.

In the consultation, mnesic errors and in naming objects were also objectified, for which it was recommended to carry out cognitive stimulation on a daily basis. In addition, he presented failures of sphincter incontinence, especially of urine and occasionally also of the anal sphincter. He had previously had episodes of myoclonus or fasciculations.

A genetic study by massive sequencing confirmed the heterozygous presence of the pathogenic variant c.1819C>T p.(Arg607Cys) in the NOTCH3 gene, a CADASIL disease.

**Conclusions:**

The anosognosia that many patients with CADASIL disease present constitutes a problem because it contributes to the delay in consultation and, therefore, the delay in the adequate diagnostic approach, therapeutic possibilities and family genetic counseling. Due in part to anosognosia, CADASIL is considered an underdiagnosed entity. Due to the lack of awareness and the consequent lack of recognition of the deficit, these people are often seen as stubborn and difficult to deal with by people in their immediate environment.

In addition, there is general difficulty in the rehabilitation process, since patients do not think the neccesity to be treated. This can generate frustration and despair both in their relatives and in the health personnel.

For all these reasons, both in anosognosia and in CADASIL disease, adequate psychological support is needed for both those affected and their families.

**Disclosure of Interest:**

None Declared

